# Meeting Report: Fourth Correlates of Protection for Next Generation Influenza Vaccines

**DOI:** 10.1111/irv.70258

**Published:** 2026-04-08

**Authors:** Alina Tscherne, Rebecca Jane Cox, Florian Krammer, Anders Bonnevie

**Affiliations:** ^1^ Ignaz Semmelweis Institute, Interuniversity Institute for Infection Research Medical University of Vienna Vienna Austria; ^2^ Influenza Centre, Department of Clinical Science University of Bergen Bergen Norway; ^3^ Department of Microbiology Haukeland University Hospital Bergen Norway; ^4^ Department of Microbiology Icahn School of Medicine at Mount Sinai New York New York USA; ^5^ Center for Vaccine Research and Pandemic Preparedness (C‐VaRPP) Icahn School of Medicine at Mount Sinai New York New York USA; ^6^ Department of Pathology, Molecular and Cell‐Based Medicine Icahn School of Medicine at Mount Sinai New York New York USA; ^7^ Ludwig Boltzmann Institute for Science Outreach and Pandemic Preparedness at the Medical University of Vienna Vienna Austria; ^8^ Department of Immunology and Transfusion Medicine Haukeland University Hospital Bergen Norway

**Keywords:** animal model, cellular immunity, correlates of protection, epidemiological modelling, influenza, influenza vaccine, mRNA vaccine, mucosal immunity, vaccine platform

## Abstract

**Background:**

This report summarizes the discussions and conclusions from the fourth “Correlates of Protection for Next Generation Influenza Vaccines” meeting, which took place in Vienna, Austria, from October 15, 2025, to October 17, 2025.

**Conclusion:**

Discussions on influenza virus correlates of protection built on prior meetings, with particular emphasis on challenges introduced by novel vaccine platforms and evolving regulatory expectations. Although established immune markers such as hemagglutination inhibiting (HI) antibodies remain central to influenza vaccine evaluation, this year's discussions highlighted the continued uncertainty and challenges around their generalizability across platforms, populations, and age groups. Regulatory perspectives emphasized the need for strong biological and clinical justification linking immune markers to protection. Overall, the meeting highlighted the importance of continued collaboration among academia, industry, and regulatory agencies to advance the identification and validation of correlates of protection, in alignment with priorities outlined in the 2025 influenza research and development road map for influenza vaccines.

## 1 Introduction

The coronavirus disease 2019 (COVID‐19) pandemic temporarily redirected a significant portion of the global scientific, medical, and development community's attention and resources toward combating severe acute respiratory syndrome coronavirus 2 (SARS‐CoV‐2). Although this shift inevitably slowed some ongoing work on other high‐impact respiratory viruses—including influenza virus—this period also offered valuable insights. The unprecedented speed and scale of developing, evaluating, and authorizing COVID‐19 countermeasures shed new light on long‐standing challenges in respiratory virus research and highlighted opportunities to modernize approaches for developing, testing, and regulating influenza medical countermeasures, particularly those based on novel technologies. As SARS‐CoV‐2 has become endemic, research efforts on influenza virus and other respiratory pathogens have resumed at levels comparable to the pre‐pandemic period, now informed by lessons learned from COVID‐19. The importance of further research on the development of effective countermeasures against influenza viruses was highlighted by recent outbreaks of influenza A virus subtypes in both humans and animals. Within only one year, the human population was facing the emergence of avian H5 in dairy cattle in the United States, severe global H5 avian influenza outbreaks [1], and the rapid spread of a mutated A/H3N2 virus (J.2.4.1, subclade K) [2].

Currently licensed seasonal influenza virus vaccines show only limited protective efficacy, and understanding influenza virus immune correlates of protection (CoPs) is crucial to develop and rapidly deploy next‐generation vaccines that are broadly protective. Although first influenza virus vaccines received approval for human use more than 60 years ago, the only universally accepted CoP for estimating influenza vaccine efficacy is the serum hemagglutination inhibition (HI) antibody titer. The HI assay is a relatively simple assay based on the capability of antibodies against the viral surface protein hemagglutinin (HA) to inhibit binding of influenza virus to red blood cells. In 1972, *Hobson et al.* found an inverse quantitative correlation between the HI antibody titers and the likelihood of infection with influenza virus [3] and defined an HI antibody titer of 1:16–1:32 to provide 50% protection from infection. In subsequent years, an HI titer ≥ 40 was defined as a surrogate of protection against influenza virus infection [4]. Although the HI assay is widely utilized across laboratories, there is a huge variation in determined HI titers between laboratories [5, 6, 7], and the assay is not ideal to measure antibody responses against influenza B strains. Furthermore, for certain influenza A virus strains, such as H5, H7, or H11 subtypes, other blood types besides those from chicken or turkey, such as horse red blood cells, are required [8, 9].

*Hobson et al.* [3] could additionally show in their experiments that other immune reactions besides the induction of HI antibody responses might be associated with protection against infection. Interestingly, individuals with no pre‐challenge influenza HI‐specific antibodies were less susceptible to infection compared to individuals with pre‐challenge influenza virus–specific antibodies, suggesting other immune mediators of protection. This is in line with recent research findings, as immune reactions independent from HI antibody titers [4], such as the activation of influenza virus–specific T cells [10], the induction of antibody responses against the neuraminidase (NA), or the induction of antibodies at mucosal sites, were associated with protection against infection. In addition, it has been shown that the HI titers are less effective in predicting protective efficacy in populations that are at high risk of developing severe influenza, such as the elderly or young children [4].

Global influenza virus experts from academia, industry, and regulatory authorities meet on a regular basis within the framework of a meeting series organized and coordinated by the International Society for Respiratory Viruses (ISRV). The fourth meeting of the “Correlates of Protection for Next Generation Influenza Vaccines” took place in Vienna, Austria, in October 2025 (program in Tables S1–S3). The ISRV “Correlates of Protection” meetings are organized globally, with the last three meetings taking place in 2010 (“Immune correlates of protection against influenza: challenges for licensure of seasonal and pandemic influenza vaccines”) [11] in Miami (USA), 2019 (“Immunological assays and correlates of protection for next‐generation influenza vaccines”) [12] in Siena (Italy), and 2023 (“Correlates of Protection for Next Generation Influenza Vaccines: Lessons Learned From the COVID‐19 Pandemic”) [13] in Seattle (USA). The meeting series aims to 1) promote international collaborations and foster knowledge exchange on CoPs, 2) discuss advances in the development of immunological assays and biomarkers, 3) update the community on regulatory and clinical implications of CoPs, 4) discuss development of influenza virus vaccines, and 5) discuss clinical and regulatory evaluation strategies [14].

During the 2025 meeting in Vienna, 158 participants from 22 countries gathered to discuss the latest advances in the prevention, treatment, and diagnosis of influenza viruses. The program comprised 13 sessions (Sessions A–L, and a dedicated poster session), featuring 39 oral presentations and panel discussions alongside 37 poster presentations. Discussions placed particular emphasis on defining CoPs for influenza viruses and exploring their potential applications across research, clinical development, and regulatory decision‐making. Significant advances have been made since the last CoPs meeting in 2023 [15], which are summarized in this report.

## 2 Main

### 2.1 Lessons From Correlates From Other Vaccines (Session A)

Regulatory authorities, including those in Europe, can license influenza vaccines based on HI antibody titer induction. Historically, an HI titer of 1:40 has been shown to provide ~50% protection against infection and has been used for decades as a surrogate CoP. However, other immune responses, such as those against the NA [15, 16] or the induction of influenza virus–specific T cells [10], provided protection against infection with influenza virus, which was independent from responses against HA. Thus, the use of an HI antibody titer of ≥ 40 as CoP, which dates back to the 1970s [3], as the sole CoP, might be outdated and should be redefined as discussed by several conference participants during the presentations and podium discussions. In the first session of this conference, the focus was on defining CoP for other pathogens, which offers the potential for utilizing these findings as a blueprint for influenza virus research. David Goldblatt (University College London, UK) provided insights into the challenges of developing and licensing efficacious pneumococcal conjugate vaccines (PCVs) against *Streptococcus pneumoniae* . The definition of an IgG anti‐capsular polysaccharide antibody titer > 0.35 μg/mL, measured by enzyme‐linked immunosorption assay (ELISA), as CoP resulted in a rapid licensure of new PCVs without prior efficacy trials and the adjustment of vaccine schedules and vaccine doses [17, 18, 19]. Furthermore, the importance of standardized assays and reagents based on internationally agreed values was highlighted, which does not only apply for PCVs but also to other pathogens, including influenza virus.

Anna Durbin (Johns Hopkins Vaccine Initiative, USA) provided an overview of the current status of vaccines against Chikungunya virus (CHIKV). Two vaccines, a live attenuated vaccine (“IXCHIQ”) and a virus‐like particle vaccine (“VIMKUNYA”), received approval for human use in the United States [20] and other countries (e.g., EU, United Kingdom, Canada, and Brazil). Notably, in August 2025, the approval for the live attenuated vaccine was suspended in the United States due to potential safety concerns, but it is still available in other jurisdictions. As discussed by Anna Durbin, there are several challenges in licensing vaccines against CHIKV. Outbreaks of CHIKV infections in the human population are not predictable and are explosive in nature. This hampers the evaluation of candidate vaccines in a phase III clinical trial, as clinical sites need to be initiated very rapidly in infection areas before the outbreak is over and larger cohorts are required to test the protective efficacy. The accepted CoP for CHIKV, derived from passive transfer of human antibodies into animals and then animal challenge, is a specific threshold of neutralizing antibodies measured by plaque reduction neutralization assay (PRNT). However, the specific antibody titer threshold is likely to be vaccine platform‐specific. As is the case for many CoPs, the measured antibody titers might not provide guidance on the durability of protection. She highlighted the importance of expanded clinical trials in which the safety and immunogenicity of the vaccines should be evaluated in older age groups (> 65 years) with comorbidities, as they are at higher risk of a severe CHIKV infection.

Ben Cowling (University of Hong Kong, China) focused on the establishment of CoPs and how to design improved clinical studies. To establish a suitable CoP, it is important to measure immune markers after vaccination and perform a detailed screening of those markers that might be of relevance to the establishment of a CoP. He suggested a randomized study design as the ideal study design, and to carefully think about the area in which the clinical trial studies are performed (e.g., high vs. low prevalence of target pathogen), as it may impact the curve for CoPs. For example, when performing a clinical trial in an area with low prevalence of the target pathogen, people are less likely to be infected with the pathogen compared to areas with high prevalence of the target pathogen, resulting in the misleading conclusion of a higher vaccine efficiency (VE). He therefore suggested performing immunogenicity substudies in very large, randomized trials with sufficient size to observe infection/disease outcomes in participants in those studies.

### 2.2 Immunology: B and T Cell Update (Session B)

Gaining insight into B and T cell responses is central to our understanding of the mechanistic basis of CoPs against influenza virus. Jenna Guthmiller (University of Colorado Anschutz Medical Campus, USA) provided an overview of recent progress in defining B cells as CoPs for influenza. She emphasized how studies on B cell subsets, repertoires, and monoclonal antibodies have advanced our understanding of serum antibodies as CoPs. In particular, she discussed how plasmablasts provide insight into the specificity of subsequent secreted antibodies following vaccination and infection [21]. She also noted that early serum antibody responses after vaccination do not necessarily predict long‐term protection, pointing out the challenge of inducing and maintaining long‐lived plasma cells capable of sustaining protective antibody levels over time [22]. In this context, a recent study showed that FcRL5^+^T‐bet^+^ memory B cells are primed for rapid recall and associated with durable vaccine responses [23]. She further discussed how new vaccine technologies, which differ in antigen presentation (e.g., soluble vs. surface‐bound) and accessibility, play a critical role in B cell activation and maturation, referencing recent work with mRNA vaccines against influenza virus and SARS‐CoV‐2 [24, 25].

This talk was followed by two short presentations. Firstly, Anette Fox (WHO Collaborating Centre for Reference and Research on Influenza Melbourne, and The Peter Doherty Institute, Australia) highlighted that vaccine‐induced B cell responses differ by type of priming and virus subtype, with H3‐HA^+^ and N2‐NA^+^ B cells being relatively expanded in infection‐primed children, whereas H1‐HA^+^ B cells were relatively expanded in vaccination‐primed children. Kayla Hanson (University of Michigan School of Public Health, USA) presented findings from a household cohort study in Nicaragua, showing that B/Victoria/2/87‐like lineage HA and NA binding antibodies function as independent CoPs, with ongoing work to estimate 50% protective thresholds.

The session then shifted focus to T cell–mediated immunity. Paul Thomas (St. Jude Children's Research Hospital, and Fred Hutch Cancer Center, USA) presented an overview and recent updates on T cells as CoPs against influenza, highlighting both CD8^+^ and CD4^+^ T cells as key contributors [26, 27]. He showed how recent advances in T cell profiling using T cell receptor (TCR) sequencing have enabled the quantification of pathogen‐specific responses with high sensitivity compared to the traditional functional T cell assays such as enzyme‐linked immunospot (ELISpot) assay or intracellular cytokine staining [28]. Complementing this, Philip Mudd (Washington University in St. Louis, USA) presented data from acutely infected humans, demonstrating robust influenza‐specific tissue‐resident CD8^+^ mucosal responses in the nasopharynx and lower airways. Finally, Carolien van de Sandt (The Peter Doherty Institute, University of Melbourne, Australia) presented data showing how CD8^+^ T cell immunity evolves across the human lifespan [29], with implications for protection against both seasonal influenza and pandemic viruses.

### 2.3 Mucosal Correlates of Immunity and Protection (Session C)

The identification of mucosal CoPs against influenza continues to be a key but complex area of research. Mucosal immunity represents the first line of defense against influenza virus infection, yet standardized methods for measuring these responses remain limited. The importance of local immune mechanisms, in particular nasal IgA, tissue‐resident memory cells, and early cytokine responses in mediating protection and reducing transmission, was discussed. New innovative approaches were presented, including work from Lena Hansen (University of Colorado Anschutz Medical Campus, USA) using spatial transcriptomics to map the organization of immune cells in the human nasal mucosa.

Charlotte Thålin (Karolinska Institute, Sweden) demonstrated the importance of local antibody responses in mediating protection against SARS‐CoV‐2 [30, 31, 32]. Longitudinal monitoring of healthcare workers demonstrated that mucosal antibodies, rather than serum markers alone, better reflected protection against re‐infection [33]. Her presentation highlighted the need for vaccines capable of eliciting strong and durable mucosal immunity, while also pointing to the challenges of measuring immune responses in the lower airways and the broader need for improved tools to define mucosal CoPs.

Building on this theme, Chris Chiu (Imperial College London, UK) discussed how controlled human infection models provide a powerful framework for defining CoPs by enabling deliberate inoculation under precisely timed and monitored conditions [34, 35, 36]. These studies have helped disentangle early mucosal and systemic immune mechanisms associated with protection, including contributions from innate pathways, dendritic cells, NK cell phenotypes, and cross‐reactive T cells. Ongoing efforts include the evaluation of aerosol‐delivered vaccines, which can boost mucosal and circulating T cell responses, as well as work by the Mucosal Immunity in Human Coronavirus Challenge (MusiCC) consortium aiming at testing mucosal vaccine platforms and refining mucosal CoP.

Adding an additional layer of complexity to understand vaccine‐induced mucosal immunity, Oliver Dibben (AstraZeneca, UK) reminded us that protection by live attenuated influenza vaccines (LAIV) hinges on efficient viral replication [37, 38]. These observations are being further investigated by the Mucosal Vaccine Evaluation (MOVE) consortium, a cross‐sector initiative aiming at understanding the mechanisms of LAIV‐induced immunity and identifying mucosal CoP to guide future vaccine design.

### 2.4 Immunological Assays (Sessions E and F)

Immunological assays are key tools to determine immune responses after natural infection and vaccination. Kanta Subbarao (Université Laval, Quebec, Canada) provided an overview of currently used methods to measure SARS‐CoV‐2 neutralizing antibody responses, such as PRNT or focus reduction neutralization test (FRNT), and how different assay platforms influence the assessment of antigenicity. She further highlighted the importance of establishing standardized assays, which would allow for a better comparison of data between different laboratories [39].

Mary Y. Wu (The Francis Crick Institute, London, UK) presented a plug‐and‐play high throughput microneutralization assay that provides a flexible system to generate high‐quality data in a short time. Due to an automated workflow, including a cell seeding machine, an automated plate washer, and an automated imager, this assay allows for the processing of 1500 samples with eight data points against one virus or 500 samples against three viruses in three days.

Maria A. Stincarelli (Vismederi, Italy) reported on the development of an antibody‐dependent cellular cytotoxicity (ADCC)–mediating antibodies assay to measure the functionality of influenza virus vaccine‐induced antibodies. Surface‐exposed proteins, such as NA, M2, or HA, have been shown to induce ADCC‐mediated antibodies, which provided a cross‐protective response against conserved influenza virus epitopes [40, 41, 42, 43]. She highlighted the importance of using ADCC‐mediated antibodies as CoPs, which may facilitate and improve the development of vaccines, but also to gain insights into immune defense mechanisms against influenza virus infections.

Felicia Hwa (The Peter Doherty Institute, University of Melbourne, Australia) focused on cross‐reactive antibody responses against Middle East respiratory syndrome‐related coronavirus (MERS‐CoV) and SARS‐CoV‐2. An increase in antibody responses against MERS‐CoV M and N proteins was observed in individuals in MERS‐CoV endemic regions and MERS‐recovered patients. Furthermore, MERS‐CoV–recovered patients had increased FcγR‐binding and IgG antibody responses against MERS‐CoV S and N proteins. In addition, SARS‐CoV‐2–immunized individuals showed increased antibody titer with cross‐reactive antibody effector functions.

Florian Krammer (Icahn School of Medicine at Mount Sinai, USA, and Medical University of Vienna, Austria) presented an overview of NA and the potential value of NA‐specific antibodies as CoPs against influenza virus infection [44, 45]. Studies in humans have shown that increased anti‐NA titers correlated with protection and reduced viral shedding/infectivity [46, 47]. NA immunity can be broad within a subtype; however, NA antibodies that provide immunity across subtypes are rare. Currently used influenza vaccines are standardized based on the HA content; thus, the NA content is very low and does not induce robust NA immunity. It will be important to undertake research in vaccine candidates that standardize their NA content in order to be able to fully evaluate the role of NA.

Arnaud Marchant (Université libre de Bruxelles, Belgium) focused on the role of non‐neutralizing antibodies as potential independent CoPs against influenza virus infection. The importance of non‐neutralizing functions of antibodies, such as the activation of complement or triggering immune responses, may vary across age groups or other population characteristics. He highlighted that the establishment of standardized assays may help to further investigate the role of non‐neutralizing antibodies against influenza virus. This may be achieved by close collaborations or within different initiatives, such as MusiCC (controlled CHIM studies aiming for investigating mucosal immunity), EVH (coordinate vaccine development for emerging viruses), or FLUCOP (standardization/development of assays to assess CoP of influenza vaccines).

Tomer Hertz (Ben‐Gurion University of the Negev, Israel) focused on the role of baseline and post‐vaccination IgA and IgG antibodies as potential independent correlates of risk (CoRs) for symptomatic influenza or COVID‐19. He proposed that IgA antibody responses are an independent CoR for symptomatic respiratory infections and suggested using both IgA and IgG markers as CoRs over individual isotypes.

The session concluded with a panel discussion about ongoing challenges in the field of performing and establishing immunological assays. The panelists highlighted the problem of non‐standardized assays, including the use of appropriate standards (e.g., mAb or pooled, convalescent sera) and the difficulties in convincing researchers to change their protocols, which they have been using in the laboratory for years/decades. Inactivated influenza virus vaccines contain at least three different viruses (A/H1N1, A/H3N2, and influenza B viruses) for which suitable and standardized assays would be required. However, this is associated with increased personnel and material costs. Also, there is a need for standardized sampling methods (e.g., nasal sampling). Regulatory authorities prefer functional antibodies over binding antibodies as their functions are better understood. However, non‐neutralizing functional antibodies are largely neglected in clinical studies.

### 2.5 Animal Models for Defining CoPs (Session G)

Animal models have been valuable tools for decades to screen vaccines or antiviral drugs before testing them in humans. Small animals, such as mice, guinea pigs, or rats, but also larger animals, such as non‐human primates and ferrets, are crucial for defining the optimal route of vaccination, testing safety and toxicity, or determining the induced immune responses. However, the overall success rate of predicting vaccine effectiveness based on data obtained from animal studies is considerably low [48, 49], highlighting the importance of advanced research in this field. In this session, the latest findings in and challenges of developing new animal models, optimizing existing models, but also establishing alternatives, such as in vitro cell culture systems, were discussed.

Stacey Schultz‐Cherry (St. Jude Children's Research Hospital, USA) provided an overview of animal models for respiratory diseases and how the priming/background immunity influences CoPs. A major problem is the limited number of suitable and standardized reagents for certain animals, such as guinea pigs, ferrets, or non‐human primates. She further highlighted the importance of choosing the right animal models, as factors such as age, weight, or animal species have an impact on the outcome of the experiments. In obese mice or ferrets [50, 51, 52], infection with influenza virus was associated with enhanced virulence, higher viral loads in the lung and heart, and an earlier and longer period of viral shedding. Furthermore, vaccinated obese mice were not protected against influenza virus infection, even when using lower viral challenge doses, higher vaccine antigen content, or passive immunization [53]. In juvenile mice, infection with influenza B virus was associated with cardiomyopathy, even when using sublethal doses. The main take‐home messages were (1) to align animal immune readouts with human vaccine trial data, (2) to define multidimensional correlates, (3) to use standardized models, (4) to bridge mechanistic and predictive correlates, and (5) to compare across different animal models.

Doris Wilflingseder (University of Veterinary Medicine Vienna, Austria) focused on in vitro models, such as organotypic airway cultures, as alternatives to animal models to evaluate mucosal immunity. Doris and her team have developed species‐specific airway barrier models, in which mucosal immune factors (e.g., resident immune cells or soluble effectors) can be integrated. This allows for studying viral entry or immune modulation of respiratory viruses in a more natural environment. Furthermore, these models have been used successfully to screen antiviral sprays against influenza virus [54] or to evaluate the neutralizing capacity of both saliva and sera of SARS‐CoV‐2–immunized individuals [55]. These in vitro models are valuable tools to study novel and innovative therapeutics and also to gain insights into pathogen entry and evasion mechanisms, although not directly translatable into clinical practice.

Florian Krammer (Icahn School of Medicine at Mount Sinai, USA, and Medical University of Vienna, Austria) provided an overview of an NA vaccine candidate and shared insights into the preclinical development of a recombinant NA‐based vaccine (N1‐MPP) [56]. NA antigenic drift is often slower than and discordant with HA antigenic drift, which would offer a potential safety net when the HA in the seasonal vaccine is mismatched with the circulating virus, and NA strain changes in the vaccine formulation may only be needed every few years. Furthermore, NA‐based vaccines offer potential cross‐protection against emerging influenza A subtypes, such as H5N1 or H9N2. In addition, mucosal immunity might play an important role in NA‐based protection [57]. The NA‐based vaccine candidate N1‐MPP contains a TLR7/8 agonist and a mucoadhesive formulation, aiming to induce an improved mucosal immune response. When given intranasally in mice, the vaccine induced serum IgG antibody responses, nasal IgA antibodies, and bronchoalveolar lavage fluid (BALF) antibody responses and induced a mucosal immune response at distinct mucosal tissues (e.g., vaginal IgA antibodies).

### 2.6 Epidemiology and Modelling of Correlates (Session H)

This session highlighted how epidemiological and statistical approaches shape our understanding of influenza CoPs (Table 1). Aubree Gordon (University of Michigan School of Public Health, USA) opened with a comprehensive assessment of cohort, household transmission, and hybrid study designs. Cohort studies offer broad generalizability and pre‐infection sampling but are limited by their differences from controlled human challenge models, including exposure heterogeneity, variable antigenic match, and ambiguity in defining endpoints such as infection, disease, or viral shedding. Household transmission studies allow intensive monitoring and efficient detection of symptomatic and asymptomatic infections, although exposure history is often uncertain and it is often difficult to identify the index case. Hybrid designs, she argued, help reconcile these limitations and have been particularly informative in identifying anti‐stalk and anti‐NA antibodies as independent CoPs [46, 58, 59, 60]. A key message was that observational studies provide powerful insights into immunity and correlates of protection, including measures such as HI titers, NA inhibiting antibodies, HA binding IgG levels, and cellular immune responses. However, as Gordon pointed out, the correlates they reveal are unavoidably shaped by variations in exposure history and immune imprinting.

**TABLE 1:** Study design, strengths, and limitations in relation to correlates of protection.

Study design	Strengths	Limitations
Randomized controlled trials	Allow causal inference; controlled exposure and endpoints	Costly; limited generalizability; ethical constraints
Observational cohorts	Reflect real‐world exposure; capture natural infection; scalable	Exposure heterogeneity; confounding by immune history; timing of sampling vs. infection
Case–control studies	Efficient for rare outcomes; use of stored samples	Selection/recall bias; challenges defining controls; uncertain endpoints
Household studies	High‐risk, well‐defined exposure; early infection capture	Small samples; variable exposure intensity
Human challenge studies	Precisely timed exposure; intensive sampling; clear infection readouts	Limited populations; restricted strains; ethical considerations

*Note:* This table summarizes key study designs and highlights their strengths and limitations based on the talks presented in this Session: “Session H: Epidemiology, Statistics and Data Science.”

Building on these methodological considerations, James Hay (Pandemic Sciences Institute, University of Oxford, UK) introduced a statistical framework to model how unobserved immunity distorts inferred CoPs relationships [61]. Through simulations that integrate exposure processes, biomarker dynamics, and study design, he showed how traditional univariable CoP curves can represent averages over unmeasured immune heterogeneity rather than true mechanistic relationships. This theme of mechanistic versus observational correlates carried into other talks, including Cheryl Cohen's (National Institute for Communicable Diseases, South Africa) comparison of molecular and serological detection of influenza virus infection in young children. Studies in a cohort with intensive three times a week PCR sampling, high infection rates, and frequent re‐infections revealed that most infections were symptomatic and that serology identified few additional infections over PCR, indicating its limited incremental utility in settings with comprehensive molecular testing [62].

Several presentations returned to the broader question of how immune markers can inform vaccine effectiveness and regulatory decision‐making. Brendan Flannery (USA) presented an application of the Prentice criteria to test‐negative vaccine effectiveness studies, showing that vaccination status, HI titers at enrollment, and infection outcomes form the expected pattern of associations, supporting the use of HI titers as surrogate endpoints while highlighting limitations when titers measured during illness differ from post‐vaccination levels.

Eva Stadler (Kirby Institute, University of New South Wales, Australia) used passive antibody transfer studies to examine the mechanistic of SARS‐CoV‐2 neutralizing antibodies to contribute to protection, showing that passive antibodies re‐capitulate vaccine‐derived protection against infection, but only partially against severe disease [63].

Yangyupei Yang (University of Michigan, USA) showed that composite SARS‐CoV‐2–specific antibody measures from multiplex assays, such as geometric mean titers or composite angiotensin converting enzyme 2 (ACE2) inhibition across circulating variants, were strongly associated with reduced Omicron infection risk and offered a robust, interpretable framework for correlates analyses.

The complementary session on clinical trials and modelling of correlates expanded on these findings by illustrating how analytical and mechanistic modelling can address limitations of observational designs. Jackie Kleynhans (National Institute For Communicable Diseases, South Africa) used data from a large household cohort study in South Africa [64] to show that integrating immunological correlates—such as pre‐season HI titers and age‐specific viral shedding duration—into transmission models enables projection of the population‐level impact of next‐generation pediatric vaccines, with simulations indicating substantial reductions in infections, hospitalizations, and deaths even under moderate vaccine effectiveness assumptions. David Hodgson (Charité Center for Global Health, Germany) presented a multidimensional immune‐modelling framework that jointly models multiple biomarkers and their kinetics, demonstrating that combining serum neutralization and mucosal IgA improves the predictive performance of CoPs across SARS‐CoV‐2 variants [65]. Together, these presentations re‐inforced a unifying message across both sessions: advancing CoPs will require approaches that combine refined study design, richer biomarker measurement, and mechanistic modelling to capture the true complexity of immunity in real‐world settings.

### 2.7 Vaccines for Pandemic Preparedness (Session I)

In terms of pandemic preparedness, the fast development and licensure of vaccines are important to combat emerging and re‐emerging pathogens. A major problem discussed during this session is how to convince people to get vaccinated during a pandemic, especially when the pandemic vaccine has been licensed swiftly, such as COVID‐19 vaccines.

Rory de Vries (Erasmus Medical Center, the Netherlands) provided an overview about the current status of vaccines against influenza virus H5 subtypes [66]. So far, more than 20 H5 vaccines, mainly based on inactivated virus preparations, have received approval for human use, with many more candidates being evaluated in clinical trials. Overall, 100s of millions of doses are stockpiled globally, mostly based on clades 1, 2.1, and 2.3.4.4b. The licensed H5 vaccines can be divided into two categories as per EMA guidance [67]: (1) vaccines for pandemic preparedness and (2) zoonotic influenza vaccines. In case of an outbreak, manufacturers can include the pandemic strain and apply for the vaccine to be authorized as a final pandemic vaccine. Examples are Foclivia (A/Vietnam/1194/2004 (H5N1), clade 1; CSL Sequirus), Adjupanrix (A/Vietnam/1194/2004 (H5N1), clade 1; GSK), or Incellipan (A/turkey/Turkey/1/2005 (H5N1), clade 2.2.1; CSL Sequirus). Zoonotic influenza vaccines are used for immunization during zoonotic outbreaks of influenza that originate from animals. Examples are Aflunov (A/turkey/Turkey/1/2005 (H5N1), clade 2.2.1; CSL Sequirus) or Celldemic (A/turkey/Turkey/1/2005 (H5N1), clade 2.2.1; CSL Sequirus). Rory further highlighted the challenges of developing vaccines against H5, which include cuts in funding (e.g., as seen for mRNA vaccines in the USA), the antigenic diversity of H5 viruses, and the relatively low HI titers, which are induced by inactivated and mRNA‐based vaccines.

Franklin R. Toapanta (University of Maryland School of Medicine, USA) presented data from a phase I clinical trial (Clinical trial: NCT05397119) in which an intranasally (IN) administered recombinant A/H5 vaccine (A/Indonesia/05/2005, clade 1) was evaluated [68]. The IN vaccination induced only low serum HI and neutralizing antibody responses. However, increased mucosal and serum IgG/IgA responses, T cell responses, and antibody‐dependent cell‐mediated cytotoxicity were detected in the study participants. Following the IM booster vaccination, participants showed increased microneutralization and HI antibody responses against different H5N1 clades.

Rebecca Cox (University of Bergen, Norway) reviewed immune correlates across influenza A subtypes, highlighting historical evidence of durable protective pre‐existing immunity. During the 2009 H1N1 pandemic, cross‐reactive H1N1pdm09 antibodies were present in older adults but not in those under 30 [69], mirroring the 1977 Russian influenza where mainly children and young adults were infected. Severe H1N1pdm09 disease was associated with low HI and MN titers, whereas higher HI titers reduced infection risk [70] and non‐viral shedders in human challenge had multifaceted immune responses including nasal HA antibodies, HI, NI, and HA‐stalk–specific IgG [71]. Overall, H1N1pdm09 vaccination preferentially recalled broadly cross‐reactive memory B cells, including those cross‐reactive with H5N1 [72]. The role of adjuvants for avian H5 and H7 candidate inactivated vaccines was emphasized, as was evidence that pandemic LAIVs, despite low HI seroconversion, effectively primed for robust recall upon subsequent inactivated pandemic vaccination (reviewed in [73]). Overall, there is a need for broader CoPs for next‐generation influenza vaccines incorporating non‐HA antigens, with tools such as ImmunoAT [74] offering a potential framework to map and predict vaccine responses.

### 2.8 Correlates Across Different Vaccine Platforms (Session J)

This session highlighted the role of CoPs with the recent advances in influenza virus vaccine platforms. Galit Alter (AstraZeneca, UK) reviewed mRNA vaccine approaches, emphasizing their flexibility, rapid update potential, and ability to induce durable germinal center responses and T cell immunity [24, 75]. She discussed enhanced strategies including chimeric/multihead HA designs, multiplexing with NA, and self‐amplifying RNA approaches. Florian Krammer (Icahn School of Medicine at Mount Sinai, USA, and Medical University of Vienna, Austria) provided an overview of other platforms, including inactivated, recombinant, live attenuated, and viral vector vaccines, highlighting differences in antibody responses, T cell induction, and production considerations.

The last two presentations in this session focused on the complementary roles of HA and NA in protection. Robert Hoelzl (Icahn School of Medicine at Mount Sinai, USA) showed that an octavalent mRNA vaccine (mRNA‐1020) including four HA strains and four matching NA strains elicited potent NA inhibiting and NA binding antibody responses not observed with traditional inactivated influenza vaccines, with titers remaining elevated above baseline at 6 months (Clinical trial: NCT05333289). Laurent Coudeville (Sanofi Vaccines, France) demonstrated through Bayesian modelling how NA antibodies can provide additional protection beyond HI titers, although the incremental benefit against non‐vaccine circulating strains remains modest.

Later that day, a panel discussion addressed regulatory challenges related to mRNA and other vaccine platforms, with Marco Calvaleri (EMA) providing the regulatory perspective. He outlined the evolution of the EMA influenza vaccine guideline, which entered into force in 2017 and is currently undergoing further revision. The guideline addresses seasonal, zoonotic, and pandemic influenza vaccines. Although primarily developed for inactivated and live attenuated vaccines, its principles are broadly applicable to newer platforms. A major shift has been the removal of the historical HI titer ≥ 40 threshold as a standalone correlate of seroprotection, recognizing that a single cutoff is unlikely to be appropriate across age groups and vaccine technologies. Greater emphasis is now placed on comprehensive immunogenicity including evaluation of anti‐NA and cell‐mediated immunity, and continuous post‐licensure effectiveness monitoring. Marco Calvaleri discussed the importance of broader functional assays, such as MN assay, and the need for clinical efficacy data when novel antigens are introduced, highlighting the ongoing effort to refine and validate CoPs for influenza vaccines.

## 3 Discussion and Summary

The fourth “Correlates of Protection for Next Generation Influenza Vaccines” meeting highlighted ongoing progress as well as persistent challenges in defining immune correlates that can support the evaluation and licensure of next‐generation influenza vaccines. Although HI antibody titers remain an established regulatory benchmark, discussions throughout the meeting reflected increasing complexity in how CoPs are interpreted and applied, particularly in the context of novel platforms such as mRNA vaccines.

A prominent focus of this year's meeting was the regulatory perspective on platform diversity and the generalizability of CoPs. Panelists (Session L) from regulatory agencies (EMA, EU; HSA, Singapore; Health Canada, Canada) emphasized that direct comparison across vaccine platforms is challenging due to differences in antigen presentation, formulation, immune mechanisms, and target populations. Even within the same platform type, individual vaccine candidates may differ in their immune profiles and in the relationship between measured immune markers and protection. As a result, a CoP validated for one vaccine or platform may not be directly transferable to another, even when comparable magnitudes of immune responses are observed.

The meeting highlighted several regulatory challenges related to CoP validation. These included the limited translatability of animal models, which may not fully capture the breadth and complexity of human immune responses, and ongoing issues with assay variability and reproducibility, particularly for cellular immune markers. In addition, current assays may not adequately quantify responses to conserved viral epitopes that could contribute to cross‐protection, complicating their use in regulatory decision‐making. Regulators emphasized the importance of a “totality of evidence” approach, integrating biological rationale, preclinical data, clinical immunogenicity, and post‐marketing evidence when evaluating novel vaccines.

The emergence of mRNA vaccines has further highlighted limitations of historical criteria used to assess influenza virus vaccines. Although HI titers and neutralizing antibodies continue to be informative, there is increasing recognition that no single immune threshold reliably predicts protection across all age groups, virus strains, and vaccine platforms. Immunobridging approaches that demonstrate a new vaccine induces immune responses comparable to or better than a vaccine with established efficacy remain an important regulatory tool, particularly when efficacy trials are not feasible. However, such approaches depend on the availability of immune markers that are strongly correlated with protection and can be measured using validated and reproducible assays and behave in the same way across different manufacturing platforms. Panel discussions involving industry and regulators debated both the opportunities and constraints associated with exploring alternative correlates, such as MN assays. Although these assays may offer additional insight where HI measurements are insufficient, their broader adoption raises concerns related to cost, standardization, and regulatory risk. At the same time, parallels were drawn to SARS‐CoV‐2 vaccine development, where neutralization assays were successfully advanced and accepted in regulatory contexts, particularly in the context of immunobridging to strain‐adapted vaccines, suggesting potential pathways forward for influenza virus.

Across the meeting, there was consensus that strengthening the scientific foundation linking immune markers to protection will be essential, particularly for next‐generation and pandemic influenza virus vaccines. Leveraging data from seasonal influenza virus studies to inform evaluations of novel platforms, together with continued collaboration among academia, industry, and regulatory agencies, was identified as a key strategy for advancing the field. These priorities align closely with the 2025 influenza research and development road map [76, 77], which highlights the identification and validation of CoPs as a central objective to guide vaccine development, regulatory decision‐making, and preparedness for emerging influenza virus threats.

## Author Contributions

**Alina Tscherne:** writing – original draft, conceptualization. **Rebecca Jane Cox:** funding acquisition, writing – review and editing, conceptualization. **Florian Krammer:** funding acquisition, writing – review and editing, conceptualization. **Anders Bonnevie:** writing – original draft, conceptualization.

## Conflicts of Interest

The Icahn School of Medicine at Mount Sinai has filed patent applications relating to SARS‐CoV‐2 serological assays, NDV‐based SARS‐CoV‐2 vaccines, influenza virus vaccines, and influenza virus therapeutics, which list F.K. as co‐inventor, and F.K. has received royalty payments from some of these patents. Mount Sinai has spun out a company, Castlevax, to develop SARS‐CoV‐2 vaccines. F.K. is co‐founder and scientific advisory board member of Castlevax. F.K. has consulted for Merck, GSK, Sanofi, Gritstone, Curevac, Seqirus, and Pfizer and is currently consulting for Third Rock Ventures and Avimex. The Krammer laboratory is also collaborating with Dynavax on influenza vaccine development.

## Supporting information


**Table S1:** Conference program—Wednesday, October 15, 2025.
**Table S2:** Conference program—Thursday, October 16, 2025.
**Table S3:** Conference program—Friday, October 17, 2025.
**Table S4:** Overview of conference sessions.

## Data Availability

The authors have nothing to report.
