# Supplementary material for: Meeting Report: Fourth Correlates of Protection for Next Generation Influenza Vaccines
Source: Influenza Other Respir Viruses. 2026 Apr 8;20(4):e70258. doi: 10.1111/irv.70258 (PMC13062645; doi:10.1111/irv.70258)
Supplement: Supplementary file 1 — Table S1: Conference program—Wednesday, October 15, 2025. Table S2: Conference program—Thursday, October 16, 2025. Table S3: Conference program—Friday, October 17, 2025. Table S4: Overview of conference sessions. [file IRV-20-e70258-s001.docx]

**Supplementary information**

**Supplementary Table 1:** Conference program- *Wednesday, October 15, 2025*

| 8:00 AM - 8:50 AM | Registration & Refreshments |
| --- | --- |
| 8:50 AM - 8:55 AM | **Welcome & Opening Address**  Florian Krammer & Rebecca Cox |
| 8:55 AM - 9:10 AM | Introduction to European Platform for Regulatory Science Research  Adam Hacker, CEPI, UK |
|  | **Session A: Lessons from Correlates from Other Vaccines**  Co-Chairs: Rebecca Cox, University of Bergen, Norway; Florian Krammer, Icahn School of Medicine at Mount Sinai and Medical University of Vienna |
| 9:10 AM - 9:40 AM | How to Evaluate Correlates and Lessons Learned from Other Vaccines David Goldblatt, UCL, London, UK |
| 9:40 AM - 10:05 AM | Challenges for Correlates of Protection and Vaccine Development for Chikungunya Anna Durbin, Johns Hopkins Vaccine Initiative, USA |
| 10:05 AM - 10:30 AM | How to Establish a Correlate and Design Better Studies Benjamin Cowling, University of Hong Kong, HK SAR, China |
| 10:30 AM - 11:00 AM | Refreshment Break |
|  | **Session B: Immunology: B and T Cell Update**  Co-Chairs: Paul Thomas, St Jude Children’s Research Hospital, USA; Jenna Guthmiller, University of Colorado, USA |
| 11:00 AM - 11:20 AM | Overview and Updates on B cells as Correlates of Protection for Influenza Jenna Guthmiller, The University of Colorado Anschutz Medical Campus, USA |
| 11:20 AM - 11:35 AM | Influenza Vaccine Responses Among Young Children first Exposed to Influenza Antigens via Infection Versus Vaccination (Paper # 24) Annette Fox, WHO CCC For Reference And Research On Influenza Melbourne At The Peter Doherty Institute, Australia |
| 11:35 AM - 11:50 AM | Humoral Correlates of Protection Against Influenza B Virus Infection (Paper # 15) Kayla Hanson, University of Michigan School of Public Health, USA |
| 11:50 AM - 12:10 PM | Overview and Updates on T cells as Correlates of Protection for Influenza Paul Thomas, St Jude Children’s Research Hospital, USA |
| 12:10 PM - 12:25 PM | Impact of Aging on CD8^+^ T-cell Immunity to Circulating and Pandemic Viruses (Paper # 1) Carolien van de Sandt, University of Melbourne At The Peter Doherty Institute, Australia |
| 12:25 PM - 12:40 PM | Robust Human Mucosal Tissue-Resident T cell Response During Acute Influenza Infection (Paper # 41) Philip Mudd, Washington University in St. Louis, USA |
| 12:40 PM - 1:40 PM | Day 1 - Lunch |
|  | **Session C: Mucosal Correlates of Immunity and Protection**  Co-chairs: Christopher Chiu, Imperial College, London; Charlotte Thalin, Karolinska Institutet, Sweden |
| 1:40 PM - 2:10 PM | Lessons Learned from Mucosal Correlates of Protection to SARS-CoV-2 Charlotte Thalin, Karolinska Institutet, Sweden |
| 2:10 PM - 2:40 PM | Role of Controlled Human Infection Model for Testing for New Vaccines and Correlates of Protection Chris Chiu, Imperial College London, UK |
| 2:40 PM - 2:55 PM | Beyond Antigenic Match: Understanding LAIV Mechanism of Action (Paper #78) Oliver Dibben, AstraZeneca, UK |
| 2:55 PM - 3:10 PM | Cross-Protective Immunity Blocking Transmission by an Intranasal Universal Influenza Vaccine  (Paper # 47) Alaura Hoag, Vivaldi Biosciences, USA |
| 3:10 PM - 3:25 PM | Tertiary Lymphoid Follicles in the Human Nasal Mucosa are Inductive sites for Humoral Immunity  (Paper # 13) Lena Hansen, University of Colorado Anschutz Medical Campus, USA |
| 3:25 PM - 3:55 PM | Day 1 - Afternoon Refreshment Break |
|  | **Session D: Oral Presentations**  Co-Chairs: Ben Cowling, University of Hong Kong, HK SAR, China; Monika Redlberger, Medical University of Vienna, Austria |
| 3:55 PM - 4:10 PM | Prior influenza vaccination shapes subsequent vaccine responses in a randomized placebo-controlled trial  (Paper # 43) Sarah Cobey, University of Chicago, USA |
| 4:10 PM - 4:25 PM | Effects of Repeated Influenza Vaccination and Infection on Durable Seroprotection in Healthcare Workers  (Paper # 29) Mai-Chi Trieu, Haukeland University Hospital, Bergen, Norway |
| 4:25 PM - 4:40 PM | A(H3N2) Antibody and B Cell Response in Repeat Compared to Naïve Vaccinees (Paper # 35) Ziheng Zhu, University of Melbourne, Australia |
| 4:40 PM - 4:55 PM | Longevity and functionality of influenza vaccine responses in young children and pregnant women in rural Bangladesh  (Paper # 23) Ingeborg Yddal, University Of Bergen, Norway |
| 4:55 PM - 5:10 PM | Quantifying Antibody Landscapes to Improve Prediction of Influenza A(H3N2) Infection and Antibody Response  (Paper # 17) Bingyi Yang, The University Of Hong Kong, HK SAR, China |
| 5:10 PM - 5:25 PM | Using Hemagglutination Inhibition Antibody Titer as Influenza Vaccine Correlate of Protection: a Systematic Review and Meta-Analysis (Paper # 12) Nancy Leung, University of Hong Kong, HKSAR, China |
| 5:25 PM - 7:15 PM | Poster Reception and Poster Presentations |
| 7:15 PM - 7:15 PM | End of Day 1 |

**Supplementary Table 2:** Conference program- *Thursday, October 16, 2025*

|  | **Session E: Immunological Assays (i)** Co-Chairs: Rebecca Cox, University of Bergen, Norway; Othmar Engelhardt, MHRA, UK |
| --- | --- |
| 8:30 AM - 9:00 AM | What are we measuring in different neutralizing antibody assays? Kanta Subbarao, Université Laval, Quebec, Canada |
| 9:00 AM - 9:25 AM | High Throughput MN Assays Mary Y Wu, The Francis Crick Institute, London, UK |
| 9:25 AM - 9:45 AM | Neuraminidase Specific Antibodies as COP Florian Krammer, Icahn School of Medicine at Mount Sinai, USA & Medical University of Vienna, Austria |
| 9:45 AM - 10:00 AM | Development of an antibody dependent cellular cytotoxicity (ADCC)-mediating antibodies assay as a method to functionally profile vaccine-induced antibodies against Influenza Viruses (Paper # 38) Maria A. Stincarelli, Vismederi, Italy |
| 10:00 AM - 10:15 AM | Divergent Humoral Landscapes: MERS-CoV-Specific Signatures and SARS-CoV-2 Cross-Reactive Antibody Effector Functions (Paper # 58) Felicia Hwa, Peter Doherty Institute, Australia |
| 10:15 AM - 10:45 AM | Day 2 - Morning Refreshment Break |
|  | **Session F: Immunological Assays (ii)** Chairs: Othmar Engelhardt, MHRA, UK; Julia Lederhofer, USA |
| 10:45 AM - 11:15 AM | Systems Serology and Non-Neutralizing Antibodies in Different Ages Arnaud Marchant, Université libre de Bruxelles (ULB), Belgium |
| 11:15 AM - 11:30 AM | Mucosal and Serum IgA are Independent Correlates of Protection for Influenza and SARS-CoV-2 Infections  (Paper # 46) Tomer Hertz, Ben-Gurion University of The Negev, Israel |
| 11:30 AM - 12:30 PM | Panel Introduction by Kanta Subbarao, Université Laval, Quebec, Canada - Immunological Assays Panelists: Othmar Engelhardt, MHRA, UK; Florian Krammer, Icahn School of Medicine at Mount Sinai, USA & Medical University of Vienna, Austria; Galit Alter, Harvard Medical School, USA; Marco Cavaleri, EMA, Amsterdam, The Netherlands; Julia Lederhofer, USA |
| 12:30 PM - 1:45 PM | Day 2 - Lunch |
|  | **Session G: Animal Models for Defining COP** Co-Chairs: Rory de Vries, Erasmus MC, Netherlands; Stacey Schultz-Cherry, St Jude Children’s Research Hospital, USA |
| 1:45 PM - 2:15 PM | Importance of Priming/Background Immunity in Animal Models and how it Influences COP Stacey Schultz-Cherry, St Jude Children’s Research Hospital, USA |
| 2:15 PM - 2:35 PM | A New Era of Respiratory Disease Modeling  (Paper # 5) Doris Wilflingseder, Ignaz Semmelweis Institute, Austria |
| 2:35 PM - 3:00 PM | Preclinical development of a recombinant NA-based vaccine Florian Krammer, Icahn School of Medicine at Mount Sinai, USA & Medical University of Vienna, Austria |
| 3:00 PM - 3:30 PM | Day 2 - Afternoon Refreshment Break |
|  | **Session H: Epidemiology, Statistics and Data Science** Co-Chairs: Aubree Gordon, University of Michigan School of Public Health, USA; Brendan Flannery, USA |
| 3:30 PM - 4:00 PM | How to Design Better Studies, Cohorts, Interventional Vaccine Studies Impact on Onward Transmission and Shedding Aubree Gordon, University of Michigan School of Public Health, USA |
| 4:00 PM - 4:15 PM | A Statistical Framework to Disentangle the Effect of Exposure History on Correlates of Protection  (Paper # 64) James Hay, Pandemic Sciences Institute, University of Oxford, UK |
| 4:15 PM - 4:30 PM | Comparing Molecular and Serological Methods for Detecting Influenza Virus Infection in Children aged 6–23 months in South Africa, 2022 (Paper # 53) Cheryl Cohen, National Institute For Communicable Diseases, South Africa |
| 4:30 PM - 4:45 PM | Prentice criteria for surrogates of protection: application to test-negative studies of influenza vaccine effectiveness  (Paper # 32) Brendan Flannery, USA |
| 4:45 PM - 5:00 PM | Recombinant Influenza Vaccines may Mitigate Attenuation Associated with Egg-Adaptation and Repeated Vaccination Against A(H3N2): Results from a Randomised Controlled Trial in Healthy Younger Adults (Paper # 34) Sheena Sullivan, Monash University, Australia |
| 5:00 PM - 5:15 PM | Using Correlates of Protection to Understand the role of Antibodies in Protection from COVID-19 (Paper # 14) Eva Stadler, Kirby Institute, UNSW Sydney, Australia |
| 5:15 PM - 5:30 PM | Design and Evaluation of Composite Antibody Measures from Multiplex Assays to Quantify Correlates of Protection Against SARS-CoV-2 (Paper # 56) Jade Yangyupei Yang, University of Michigan, USA |
| 5:30 PM - 5:30 PM | End of Day 2 |
| 7:00 PM – 11:00 PM | Conference Dinner at Luftburg Kolarik im Prater |

**Supplementary Table 3:** Conference program- *Friday, October 17, 2025*

| 8:15 AM - 8:45 AM | Keynote - Use of Immune Markers for Regulatory Decisions: Where do we stand with Influenza Vaccines? Marco Cavaleri, EMA, Amsterdam, The Netherlands |
| --- | --- |
|  | **Session I: Vaccines for Pandemic Preparedness** Co-Chairs: Galit Alter, Harvard Medical School, USA; Stephen Mark Tompkins, University of Georgia, USA |
| 8:45 AM - 9:15 AM | Overview of H5 Vaccines Rory de Vries, Erasmus MC, The Netherlands |
| 9:15 AM - 9:30 AM | An intranasal adjuvanted, recombinant influenza A/H5 vaccine primes against diverse H5N1 clades: a phase I trial  (Paper # 77) Franklin R. Toapanta, University of Maryland School of Medicine, USA |
| 9:30 AM - 9:50 AM | Correlates Across Subtypes Rebecca Cox, University of Bergen, Norway |
| 9:50 AM - 10:25 AM | Challenges in Correlates for Pandemic Vaccines - Panel Discussion Panelists: Marco Cavaleri, EMA, Amsterdam, The Netherlands; Kanta Subbarao, Université Laval, Quebec, Canada; Stacey Schultz-Cherry, St Jude Children’s Research Hospital, USA; Rory de Vries, Erasmus MC, The Netherlands |
| 10:25 AM - 10:55 AM | Day 3 - Morning Refreshment Break |
|  | **Session J: Correlates Across Different Vaccine Platforms** Co-Chairs: Alessandro Lazdins, CEPI, UK; Kanta Subbarao, Université Laval, Canada |
| 10:55 AM - 11:25 AM | Overview of mRNA Platforms for Vaccine Development Galit Alter, Harvard Medical School, USA |
| 11:25 AM - 11:55 AM | Overview of Other Vaccine Platforms Florian Krammer, Icahn School of Medicine at Mount Sinai, USA & Medical University of Vienna, Austria |
| 11:55 AM - 12:10 PM | Potent Neuraminidase-Inhibiting and Binding Antibody Responses Elicited by a an Octavalent mRNA Influenza Vaccine (containing both HA and NA) in a Phase 1/2 Clinical Trial  (Paper # 45) Robert Hoelzl, Icahn School Of Medicine, USA |
| 12:10 PM - 12:25 PM | Correlate of Protection for Influenza: Complementarity Between Hemagglutinin (HA) and Neuraminidase (NA)  (Paper # 73) Laurent Coudeville, Sanofi, France |
| 12:25 PM - 1:25 PM | Day 3 - Lunch |
|  | **Session K - Oral Presentations on Clinical Trials and Modelling of Correlates** Co-Chairs: Sheena Sullivan, Monash University, Australia; Stephen Mark Tompkins, University of Georgia, USA |
| 1:25 PM - 1:40 PM | Immune profiling uncovers correlates of severity in multisystem inflammatory syndrome in children (MIS-C) (Paper # 21) Robert Mettelman, St Jude Children's Research Hospital, USA |
| 1:40 PM - 1:55 PM | Revealing Dose and Repeat Effects of a Novel Influenza Vaccine on Humoral Immunity (Paper # 3) Ludivine Grzelak, Peter Doherty Institute, Australia |
| 1:55 PM - 2:10 PM | Joint Bayesian Modelling of H1N1 and H3N2 Antibody Dynamics Clarifies Co-Infection and Cross-Reactivity  (Paper # 20) Tim Tsang, The University Of Hong Kong, HK SAR, China |
| 2:10 PM - 2:25 PM | Integrating Immunological Correlates into Transmission Modeling to Project the Impact of Next-Generation Pediatric Influenza Vaccines in South Africa (Paper # 16) Jackie Kleynhans, National Institute For Communicable Diseases, South Africa |
| 2:25 PM - 2:40 PM | Correlates of Protection Through Multidimensional Immune Modelling across Respiratory Viruses (Paper # 27) David Hodgson, Charité, Universitätsmedizin Berlin, Germany |
| 2:40 PM - 3:10 PM | Day 3 - Afternoon Refreshment Break |
|  | **Session L - Perspectives on Regulatory Challenges to mRNA and Other Platforms** Co-Chairs: Adam Hacker, CEPI, UK; Marco Cavaleri, EMA, The Netherlands |
| 3:10 PM - 3:55 PM | Perspectives on Regulatory Challenges to mRNA and Other Platforms Panellists: Anuradha Poonepalli, HSA, Singapore; Charlene Young, HC, Canada, Leonoor Wijnans, College Ter Beoordeling Van Geneesmiddelen, The Netherlands |
| 3:55 PM - 4:45 PM | What is the Current View on new Vaccine Platforms - Panel Debate Moderated by Marco Cavaleri, EMA, Amsterdam, The Netherlands Panellists: Alan Embury, Moderna; Annaliesa Anderson, Pfizer; Jean-François Toussaint, Sanofi |
| 4:45 PM - 5:00 PM | Closing Remarks Florian Krammer |

**Supplementary Table 4:** Overview of Conference Sessions

| **Session** | **Title** |
| --- | --- |
| **A** | Lessons from Correlates from Other Vaccines |
| **B** | Immunology: B and T Cell Update |
| **C** | Mucosal Correlates of Immunity and Protection |
| **D** | Oral Presentations |
| **E** | Immunological Assays (i) |
| **F** | Immunological Assays (ii) |
| **G** | Animal Models for Defining Correlates of Protection |
| **H** | Epidemiology, Statistics and Data Science |
| **I** | Vaccines for Pandemic Preparedness |
| **J** | Correlates Across Different Vaccine Platforms |
| **K** | Oral Presentations on Clinical Trials and Modelling of Correlates |
| **L** | Perspectives on Regulatory Challenges to mRNA and Other Platforms |

*Note. Summary of all scientific sessions presented at the meeting*
